# Supplementary material for: Positive Impact of Home ERT for Mucopolysaccharidoses and Pompe Disease: The Lesson Learnt from the COVID-19 Pandemic
Source: Healthcare (Basel). 2023 Apr 19;11(8):1176. doi: 10.3390/healthcare11081176 (PMC10137846; doi:10.3390/healthcare11081176)
Supplement: Supplementary file 1 [file healthcare-11-01176-s001.zip › healthcare-2238985-supplementary.pdf]

Home ERT – Evaluation of satisfaction degree in patients with lysosomal storage diseases.

Name (initials)
Age
Sex
School degree
Diagnosis

1) Referring to your health concern, did you choose to undergo infusion therapy
    a. At home
    b. At the Referral Centre

2) Are you satisfied about your choice?
    a. Yes
    b. No
    c. Not sure

3) How do you rate your satisfaction being infused at home or at the center (choose the closest degree)
    a. Not at all satisfied
    b. Quite unsatisfied
    c. A little bit unsatisfied
    d. Neither satisfied nor unsatisfied
    e. A little bit satisfied
    f. Quite satisfied
    g. Totally satisfied

    Please motivate your answer
    ……
    ……
    ……

4) Since you decided, did you experience any benefit / changes?
    a. Yes
    b. No
    c. Not sure

5) Please, specify the kind of changes (you can choose multiple answers)
    a. Physical
    b. Psychological
    c. Familial relationship

6) How do you rate these changes?
    a. Totally negative
    b. Quite negative
    c. A little bit negative
    d. Neither negative - nor positive
    e. A little bit positive
    f. Quite positive

g. Totally positive

Please motivate your answer

……
……
……

7) How do you evaluate the organization put in place by the Centre to allow Home Ert?
   a. Unsatisfactory
   b. Sufficient
   c. Discrete
   d. Good
   e. Excellent

   Please motivate your answer

   ……
   ……
   ……

8) Did you appreciate the psychological help offered by the Centre both in presence and on line?
   a. Yes
   b. No
   c. Indifferent

9) If you answered Yes to the previous question, how much did you appreciate this psychological help?
   a. Enterely unsatisfied
   b. Quite unsatisfied
   c. A little bit unsatisfied
   d. Indifferent
   e. A little bit satisfied
   f. Quite satisfied
   g. Enterely satisfied

10) Did you experience any change/benefit thanks to the psychological help?
    a. Yes
    b. No
    c. Not sure

11) Which kind of changes did you experience? (you can make multiple choises)
    a. Psychological
    b. Phisical
    c. Familial relationship

12) How do you rate the quality of these changes?
    a. Totally negative
    b. Quite negative
    c. A little bit negative
    d. Nor negative or positive

e. A little bit positive
f. Quite positive
g. Totally positive

Please motivate your answer
……
……
………..

13) How are you psychologically feeling at the current moment? How do you assess your health conditions?
………
………..
……….

14) Considering your actual health and the still present pandemic, which would your feeling be in the event of a new lockdown?
a. Resignation
b. Anxiety
c. Fear
d. Distress
e. Surprise
f. Challenge
g. Optimism

15) Have you made projects for your near future?
……
……
……

16) How do you foresee your near future?
……
……
……

17) What would you improve in the assistance and care of the Centre and its clinical staff?
……
……
……
